# Strong association between pseudogenization mechanisms and gene sequence length

**DOI:** 10.1186/1745-6150-4-38

**Published:** 2009-10-06

**Authors:** Amit N Khachane, Paul M Harrison

**Affiliations:** 1 Department of Biology, McGill University, Stewart Biology Building, 1205 Docteur Penfield Ave, Montreal, QC, H3A 1B1, Canada

## Abstract

**Abstract:**

Pseudogenes arise from the decay of gene copies following either RNA-mediated duplication (processed pseudogenes) or DNA-mediated duplication (nonprocessed pseudogenes). Here, we show that long protein-coding genes tend to produce more nonprocessed pseudogenes than short genes, whereas the opposite is true for processed pseudogenes. Protein-coding genes longer than 3000 bp are 6 times more likely to produce nonprocessed pseudogenes than processed ones.

**Reviewers:**

This article was reviewed by Dr. Dan Graur and Dr. Craig Nelson (nominated by Dr. J Peter Gogarten).

## Background

Pseudogenes are defective copies of genes that evolve neutrally. Pseudogenes originating from protein-coding genes lack the ability to code for proteins and bear features of coding sequence decay, such as: *i) *the presence of premature stop codon/frameshift mutations, *ii) *nonsynonymous/synonymous (Ka/Ks) substitution rates of ~1.0, and *iii) *truncation of protein domains. Pseudogenes are classified basically into two types: *i) *'Processed' or retrotransposed pseudogenes, which arise following a RNA-mediated duplication (retrotransposition) [1-3] and, *ii) *'Nonprocessed' pseudogenes, which arise following a DNA-mediated duplication [4]. Unlike nonprocessed pseudogenes, gene copies that arise following retrotransposition do not retain promoter regions of their parent genes. These copies are generally considered to be functionless at the time of birth ('dead on arrival') [1,3]. Some of these, over the time, are able to recruit new promoters to become functional [5,6]. Hence, in this study, we considered retrotransposition as a distinct pseudogenization mechanism.

An intriguing and a basic aspect that remains yet unknown is whether sequence length plays any role in the evolution of pseudogenes. If so, is such an effect common to both basic categories of pseudogenes (*i.e*., processed and nonprocessed)? Here, we addressed this question for the annotated pseudogenes of processed and nonprocessed categories from the human and mouse genomes.

## Results and Discussion

**Figure 1:**
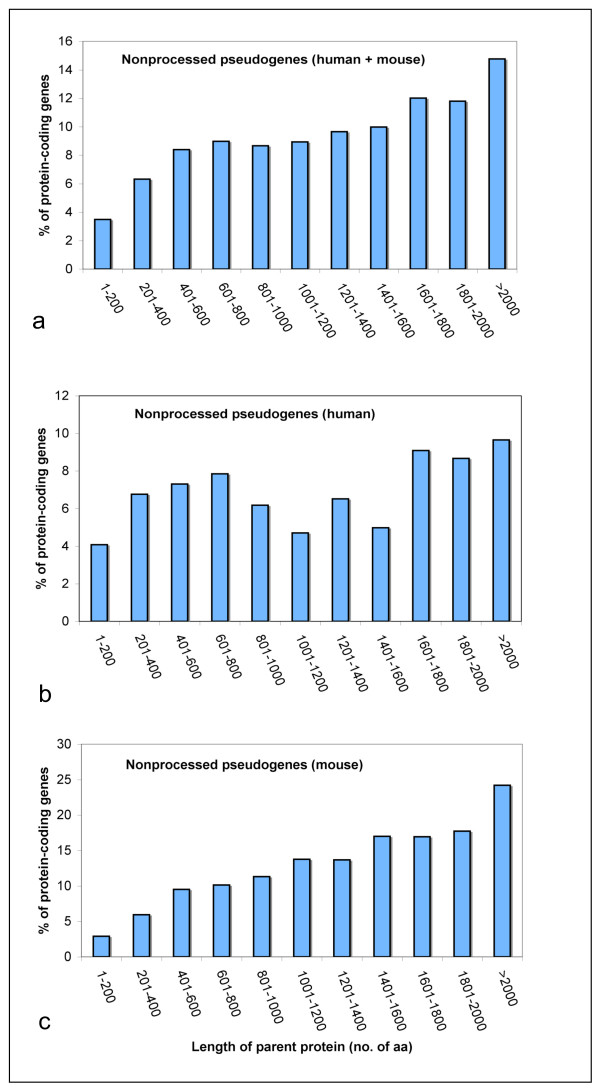
**Percentage of protein-coding genes producing nonprocessed pseudogenes in the various length categories**. (a) For human and mouse combined, (b) for human, and (c) for mouse.

**Figure 2:**
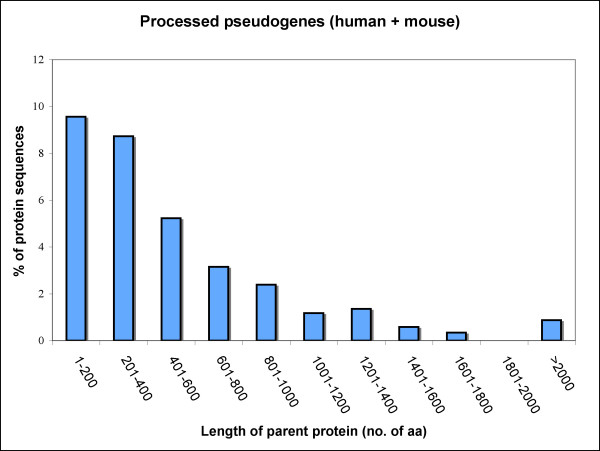
**Percentage of parental proteins (human+mouse) associated with processed pseudogenes (retropseudogenes) in the various length categories**.

The proportion of protein-coding genes that produced nonprocessed pseudogenes was found to increase with parental gene length (Fig. 1) with an unexplainable decrease in the mid-range in human (Fig. 1b), suggesting that following a DNA-mediated duplication event, longer protein-coding genes are generally more likely to become pseudogenes than their shorter counterparts. In contrast, the proportion of protein-coding gene transcripts that produced processed pseudogenes was found to decrease with sequence length (Fig. 2), which is in agreement with an earlier report that found that reverse-transcribed gene copies in human are of shorter length [3]. The trend in the category of processed pseudogenes is the same for human and mouse genomes when analyzed separately (data not shown). Within the processed pseudogene category, only 67 cases (human and mouse combined) have parental gene length >1000 amino acids (aa), whereas 421 in the case of nonprocessed category, suggesting that longer protein coding genes are ~6 times more likely to produce nonprocessed pseudogenes than processed ones.

These trends are explainable as follows. Under a neutral evolutionary scenario, longer sequences are more likely to accumulate deleterious mutations than shorter ones. This seems to be the case in nonprocessed pseudogenes. A similar effect has been noticed in protein-coding genes associated with hereditary diseases [7]. In the case of retropseudogenes, additional evolutionary forces seem to play a role. This may have to do with the higher propensity of shorter genes to undergo retrotransposition [3]. Because the probability of interruption in the transcription of parent genes and subsequent reverse transcription during a retrotransposition event is higher for longer genes than for shorter ones, we anticipate seeing a larger proportion of successfully retrotransposed sequences to evolve from shorter genes. The abundance of transcripts may also influence the number of retropseudogenes arising from a gene. It has been shown that genes with retropseudogenes tend to be expressed in several tissues and generally do not tend to be tissue-specific [3].

## Conclusion

This study demonstrates that the occurrence of pseudogenized gene copies is a function of gene length. Parental genes encoding for proteins longer than 1000 aa are 6 times more likely to produce nonprocessed pseudogenes than processed ones.

## Methods

The annotations of pseudogenes were obtained from pseudogene.org [8] on November 2007, human proteins from ENSEMBL release 47  and mouse proteins from ENSEMBL release 31 (that was also used for the annotation of pseudogenes). The total number of sequences in each category is as follows: human nonprocessed pseudogenes (1494), human processed pseudogenes (2858), human proteins (47550) and human protein-coding genes (23944); mouse nonprocessed pseudogenes (1753), mouse processed pseudogenes (2393), mouse proteins (31535) and mouse protein-coding genes (24461). The number of nonprocessed pseudogenes in each length category was normalized by the number of protein-coding genes, whereas in the case of processed pseudogenes, by the number of transcript/protein sequences because the transcripts act as direct precursors for the birth of retrotransposed copies.

## Competing interests

The authors declare that they have no competing interests.

## Authors' contributions

ANK performed the analyses. ANK and PMH interpreted the results and wrote the paper.

## Reviewers' comments

### Reviewer's report 1

Dr. Dan Graur

Department of Biology and Biochemistry, University of Houston, USA

Accepted for publication with some stylistic suggestions (not for publication).

### Reviewer's report 2

Dr. Craig Nelson (nominated by Dr J Peter Gogarten, University of Connecticut).

Molecular & Cell Biology, University of Connecticut, USA.

In this study the authors describe a relationship between the protein coding length of a gene and number of RNA and DNA mediated duplicate pseudogenes derived from each gene. They find that long genes tend to produce fewer RNA-generated pseudogenes than do shorter genes.

The data presented appears sound and the core finding valid. I recommend accepting for publication following minor revision.

Several suggestions follow for possible improvements to the manuscript.

Major suggestions:

1) Both RNA-mediated and DNA-mediated duplication events give rise to duplicate genes that may become pseudogenized over time. Referring to DNA-mediated events as duplications and RNA-mediated events as something other than duplications does not reflect this fact. I urge the authors to change the way this is presented in the text.

***Author's response: ***For the sake of clarity, we have now introduced the above suggested terms.

Unlike gene copies that arise from DNA-mediate duplication, copies that arise following a RNA-mediated duplication or retrotransposition are essentially functionless at the time of birth, because they do not retain the parental promoter regions for their immediate transcription. Hence, in this study, we considered retrotransposition as a distinct event generating retrotransposed pseudogenes (retropseudogenes). Only some of the retrotransposed copies are able to recruit new promoters over the time to become functional.

2) "Processed" and "Non-processed" are not intuitive terms for those outside the field and, while these terms are correct, I suggest that the authors adopt more descriptive terms like RNA-mediated and DNA-mediated duplications, and/or retrotransposed pseudogenes.

***Author's response: ***We have now refined the text to make it more understandable.

3) No clear distinction I made between the duplication event and the pseudogenization event. Some discussion about which of these events are detected and analyzed by the authors and what impact this might have of the core finding would be welcome.

***Author's response: ***We have discussed the above issue in the Results and Discussion section (second paragraph). Also, refer to response to comment 1. We considered RNA-mediated duplication (retrotransposition) *per se *as an event contributing to the birth of pseudogenes. In this work, we were interested in studying whether sequence length plays any role in the evolution of the two distinct classes of pseudogenes.

4) In Materials and Methods section, more specific data sources and preprocessing methods should be specified. For example, which Ensembl release was used, and was any filter for pseudogenes and protein coding genes applied? The numbers of pseudogenes and protein-coding genes in the paper are quite different from the pseudogene data from Pseudogene.org and the protein-coding genes from Ensembl.org. For example, the protein-coding genes listed in Ensembl (release 55) are around 22,000 but the number in the text is 46,689.

***Author's response: ***We have now mentioned the Ensembl release number in the Methods section. We downloaded pseudogene data in November 2007 from Pseudogene.org. The site has been recently updated. In the pseudogene.org database, some pseudogenes are marked as 'unclassified', note that we have included only pseudogenes that are annotated as processed and nonprocessed pseudogenes.

The figure 46,689 is for the number of human proteins (< = 2000 aa). We have now included cases with sequence length >2000 aa and have corrected the number in the text accordingly.

5) Is the trend same with human and mouse genomes analyzed separately? Any reason to put them together?

***Author's response: ***Individually, they show similar trends, rising percentage values with increasing sequence length in the case of nonprocessed pseudogenes (Fig 1) and falling percentage values with increasing sequence length in the case of processed pseudogenes (Fig 2).

Minor suggestions:

1) From the figures, it is not easy to see that the longer parental genes (>1000 AAs) are 6 times more prone to produce non-processed pseudogenes than processed. Figure or Table might help the cause.

***Author's response: ***We have now discussed the above in the text.

2) In Fig 1 and 2, are there any parental genes longer than 2000AA? And

corresponding pseudogenes?

***Author's response: ***Yes, there are. We have now included them in the analysis.

3) In the discussion the authors mention: "Because the probability of interruption in

the transcription of parent genes and subsequent reverse transcription during a retrotransposition event is higher for longer genes than for shorter ones". It might be worth mentioning here that transcript abundance also has a large effect on this probability and that transcript abundance, gene length, and the abundance of retrotransposed pseudogenes are all correlated.

***Author's response: ***We agree with the comments and have included them in the discussion.
